# Health Tract.—No. 61

**Published:** 1862-02

**Authors:** 


### HEALTH TRACT.—NO. 61.

### FAILING EYESIGHT.

THE sight begins to fail from forty to fifty, by an instinctive preference of larger print; a seat near the window for reading is unwittingly selected; there is an effort to place the paper at a convenient distance from the eye, or to turn it so as to get a particular reflection of the light; next the finger begins to be placed under the line read, or there is a winking of the eye as if to clear it, or a looking away at some distant object to rest it; or the fingers are pressed over the closed lids in the direction of the nose, to remove the surplus water caused by straining. When spectacles are first used, they should not be kept on steadily, only in the early morning, or cloudy day, cr dim light, or with fine print, or sewing. Favor the failing sight as much as possible:

1st. By sitting in such a position as will allow the light to fall upon the page or sewing obliquely over the shoulder.

2d. By not using the eyes for such purposes by any artificial light, or before sunrise, or after sunset.

3d. By avoiding the special use of the eyes in the morning before breakfast.

4th. By resting them for half a minute or so, while reading or sewing, or looking at small objects, by looking at things at a distance or up to the sky, relief is immediately felt by so doing.

5th. Never pick any collected matter from the eye-lashes or corners of the eyes with the finger-nails; rather moisten it with the saliva and rub it away with the ball of the finger.

6th. Frequently pass the balls of the fingers over the closed eyelids, towards the nose; this carries off any excess of water into the nose itself by means of the little canal which leads into the nostril from each inner corner of the eye, which canal tends to close up in consequence of the slight inflammation which attends weakness of eyes.

7th. Keep the feet always dry and warm, so as to draw any excess of blood from the other end of the body.

8th. Use eye-glasses at first, carried in the vest-pocket, attached to a guard, for they are instantly adjusted to the eye with very little trouble; whereas, if common spectacles are used, such a process is required to get them ready, that to save trouble, the eyes are often strained to answer a purpose.

9th. Wash the eyes abundantly every morning. If cold water is used, let it be flapped against the closed eye with the fingers of the hand, not striking hard against the balls of the eyes. But it would seem a better plan to open the eyes in pure warm water, because warm water is more penetrating than cold; it dissolves much more readily and rapidly any hardened matter that may be about the lids, and is more soothing and more natural.

10th. The moment the eyes feel tired, the very moment you are conscious of an effort to read or sew, lay aside the book or needle, and take a walk for an hour, or employ yourself in some active exercise not requiring the close use of the eyes.

